# Compound double ileoileal and ileocecocolic intussusception caused by lipoma of the ileum in an adult patient: A case report

**DOI:** 10.1186/1752-1947-5-452

**Published:** 2011-09-12

**Authors:** Avdyl S Krasniqi, Astrit R Hamza, Lulzim M Salihu, Gazmend S Spahija, Besnik X Bicaj, Selvete A Krasniqi, Fisnik I Kurshumliu, Lumturije H Gashi-Luci

**Affiliations:** 1 Department of Surgery, University Clinical Centre of Kosova, Rrethi Spitalit street, pn.; 10 000, Prishtina, Kosovo; 2 Faculty of Medicine, University of Prishtina, Rrethi Spitalit street, pn.; 10 000, Prishtina, Kosovo

## Abstract

**Introduction:**

The initial diagnosis of intussusception in adults very often can be missed and cause delayed treatment and possible serious complications. We report the case of an adult patient with complicated double ileoileal and ileocecocolic intussusception.

**Case presentation:**

A 46-year-old Caucasian man was transferred from the gastroenterology service to the abdominal surgery service with severe abdominal pain, nausea, and vomiting. An abdominal ultrasound, barium enema, and abdominal computed tomography scan revealed an intraluminal obstruction of his ascending colon. Plain abdominal X-rays showed diffuse air-fluid levels in his small intestine. A double ileoileal and ileocecocolic intussusception was found during an emergent laparotomy. A right hemicolectomy, including resection of a long segment of his ileum, was performed. The postoperative period was complicated by acute renal failure, shock liver, and pulmonary thromboembolism. Our patient was discharged from the hospital after 30 days. An anatomical pathology examination revealed a lipoma of his ileum.

**Conclusions:**

Intussusception in adults requires early surgical resection regardless of the nature of the initial cause. Delayed treatment can cause very serious complications.

## Introduction

Intussusception was reported for the first time in 1674 by Barbette of Amsterdam. Intussusception, or 'introsusception' as it was named then, was later detailed in 1789 by John Hunter [1]. In 1871, Sir Jonathan Hutchinson was the first to successfully operate on a child with intussusceptions [2]. Intussusception is relatively frequent in children but is rare in adults [3]. Adult intussusception represents 1% of all bowel obstructions and 5% of all bowel intussusceptions [4]. In contrast to pediatric intussusception, which is idiopathic in 90% of cases, adult intussusception has an organic lesion in 70% to 90% of cases [5]. Adult intussusception can present with atypical symptoms of an acute, subacute, or chronic clinical entity, and timely diagnosis is often missed, leading to a delay in proper treatment [3]. Although it is generally accepted that adult intussusception requires surgical resection because of the underlying pathology in the majority of patients, the extent of resection and the question of whether the intussusception should be reduced remain controversial [6]. The aim of this report is to present a rare case of double ileoileal with ileocecocolic intussusception in an adult patient. The case was caused by the submucosal lipoma of the ileum and resulted in serious complications due to delayed surgical treatment.

## Case presentation

**Figure 1:**
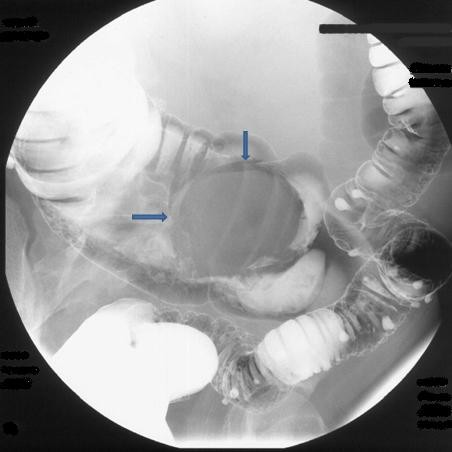
**A barium enema image of the colon shows a filling defect in the ascendant colon (arrows)**.

**Figure 2:**
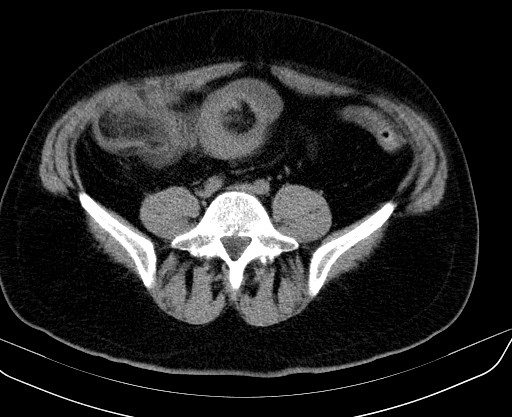
**An abdominal computed tomography scan shows a 'sausage'-shape soft-tissue mass in the ascendant colon and thickened walls of the ileum**.

**Figure 3:**
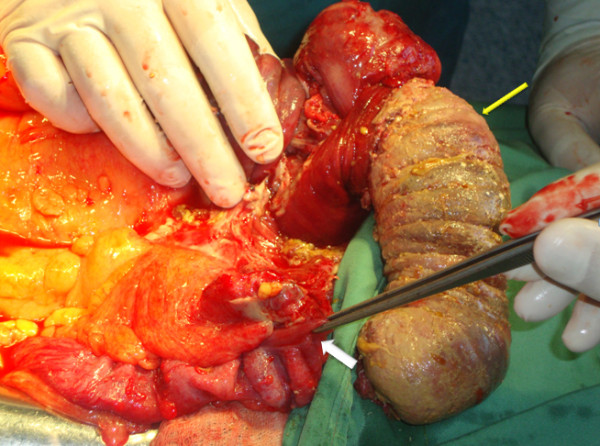
**A double intussusception of the ileum after desinvagination from the ascendant colon (thick arrow) and necrotic change in the wall of the ascendant colon (thin arrow)**.

A 46-year-old Caucasian man was transferred from the gastroenterology service to the abdominal surgery division for intractable severe abdominal pain accompanied by nausea and vomiting. He had a four-month history of abdominal discomfort, namely intermittent abdominal cramping pain of mild to moderate severity in his middle and lower quadrants. His medical history was unremarkable. A review of his systems revealed weight loss of nine pounds during the previous three months. Eight days earlier, he had been admitted to the gastroenterology service for a diagnostic work-up and medical treatment. During the initial physical examination, he appeared in good general condition, was normothermic, and had a slightly distended abdomen, which, however, was soft and non-tender. No rebound effect was elicited. A rectal examination revealed no masses or blood. Laboratory results were all within normal range. An abdominal ultrasound showed a hyperechoic mass in his ileocecal region. A barium enema showed an oval-shape filling defect in his ascending colon (Figure 1). An abdominal computed tomography (CT) scan showed an irregular 'target' and a 'sausage'-shape soft-tissue mass with thickened walls of his cecum and terminal ileum (Figure 2). Although all diagnostic procedures clearly suggested colonic obstruction, our patient refused transfer to the surgery department until the pain, nausea, and vomiting became persistent and more severe. During his admission to surgery, plain abdominal films clearly demonstrated signs of intestinal obstruction, air-fluid levels in his small intestine, and the absence of air in his colon. Our patient underwent an emergent median laparotomy. During the operation, a large intussuscepted mass was found. It was located in the region of his ascending colon and hepatic flexure, into which a large segment of his ileum, appendix, cecum, and part of his ascending colon were invaginated. Because of compromised perfusion and swelling of his colonic wall and because of an unsuccessful attempt at manual desinvagination, a round incision in his ascending colon was made, and his invaginated cecum and terminal ileum were pushed backward with the intention of preserving as much viable small bowel as possible. An *in situ *macroscopic view showed that a 15 cm segment of his ileum was intussuscepted into the distal 20 cm of his terminal ileum, which, together with his appendix and cecum, subsequently intussuscepted into his ascending colon, resulting in a double ileoileal and ileocecocolic intussuception. His cecum and about 30 cm of his terminal ileum were entrapped in the intussuscipiens and had necrotic changes in their walls (Figure 3). A right hemicolectomy that included an approximately 40 cm segment of his ileum was performed. The continuity of the digestive tube was reestablished by primary single-layer end-to-end ileotransverse anastomosis with 3.0 polydioxanone sutures.

**Figure 4:**
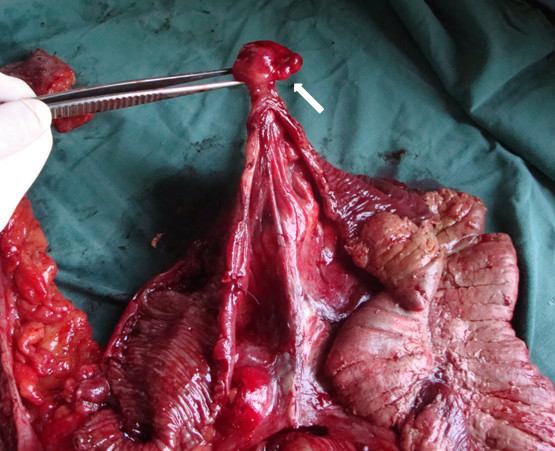
**A pendulant polipoid submucosal tumor of the terminal ileum served as a lead point for the intussusception**.

**Figure 5:**
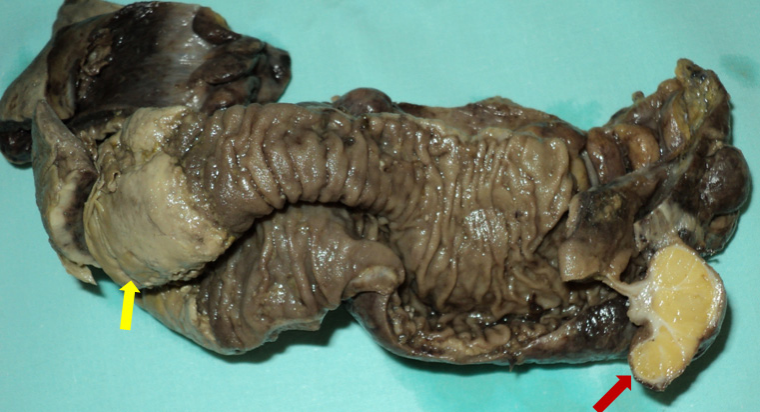
**A specimen fixed in formalin shows a submucosal pendulant lipoma (3.5 cm in diameter) that after a histopathology examination was revealed to be benign**.

The macroscopic examination of the specimen identified a 4 cm pendulant polypoid mass in his terminal ileum (Figure 4). An anatomical pathology examination of the resected specimen revealed a submucosal tumor of his ileum about 3.5 cm in diameter with features of a benign lipoma (Figure 5).

The postoperative course was eventful. As a result of toxic syndrome (probably due to protracted preoperative intestinal obstruction and delayed surgical treatment), the postoperative period was complicated by high fever (39.5°C), hypotension, acute renal failure within the first six postoperative hours, and significant abnormalities of liver function tests on the first postoperative day. Multiorgan failure ensued, and our patient was transferred to the intensive care unit. Renal failure resolved after hemodialysis sessions carried out each day for one week. On the twentieth postoperative day, the patient developed all clinical manifestations of pulmonary embolism which was treated with heparin initially, and subsequently with warfarin. On the 30th postoperative day, our patient was discharged from the hospital in good condition.

## Discussion

Intussusception remains a rare condition in adults, representing 1% of bowel obstructions or 0.003% to 0.02% of all hospital admissions [3]. In contrast to pediatric intussusception (which is mainly of unclear etiology), adult intussusception in 90% of cases is secondary to an organic lesion within the bowel wall [7-10]. Although the mechanism of development is unknown, it is believed that any lesion in the intestinal wall or irritant within the lumen which alters normal peristalsis is able to initiate an invagination [7,11]. There are different classification systems of intussusceptions. In general, intussusception is classified as enteric or colonic according to the location of the pathologic lead point [12]. The enteric group includes jejunojejunal, ileoileal, and ileocolic intussusceptions, whereas the colonic group includes ileocecal-colic, colocolonic, sigmoidorectal, and appendicicocecal intussusceptions. Ileocolic and ileocecal-colic intussusceptions are distinguished by the site of the pathologic lead point. In ileocolic intussusception the lead point is in the ileum, but in ileocecal-colic intussusception the lead point is in the ileocecal valve. However, in clinical practice, it is difficult to differentiate some of the complicated advanced forms of ileocecal-colic intussusceptions [13]. In the present case, although the intussusception was ileocecal-colic, the initial pathologic lead point was located in the ileum and caused the double ileoileal intussusception (Figure 3). Then the double ileoileal intussusception continued to act as a lead point through the cecum toward the ascending colon, thus causing ileocecal-colic intussusceptions. A similar case with double invagination of the ileum was reported by Constanzo and colleagues [14] (2007).

Adult intussusception presents with a variety of non-specific symptoms that can have an acute, intermittent, or chronic course. Since only about 9% to 10% of adult intussusceptions present with the typical triad of abdominal pain, palpable abdominal mass and bloody stool, the preoperative diagnosis is usually very difficult [7].

Early and accurate diagnosis is essential because a delay can lead to intestinal ischemia, perforation, and peritonitis and result in a potentially fatal outcome [15-17]. A number of different diagnostic methods - such as CT scan, barium imaging, abdominal ultrasound, endoscopic examination, and angiographic and radionucleotide studies - have been described as useful in the diagnosis of intussusceptions [18,19]. The abdominal CT scan has been proven to be the most useful diagnostic method, and ultrasound is the second most accurate; both reveal a characteristic 'target' or 'sausage'-shape mass. In our case, the abdominal CT scan, done nine days before transfer to surgery, showed a characteristically laminated 'target mass' in the ileocecal region (Figure 2); however, the abdominal pain and accompanying symptoms did not correlate with the severity of the radiological findings. Because our patient was not willing to undergo surgical treatment at this stage, the gastroenterology team performed a barium enema examination aiming at both diagnostic and therapeutic effects. However, this procedure yielded no therapeutic results in terms of reduction. This confirmed the findings of other authors [9,15], who reported that barium studies, despite good diagnostic and therapeutic effects in children with presumed diagnosed intussusception, do not have any considerable hydrostatic reducing effect in adults, because of the high incidence of underlying anatomical abnormalities.

The treatment of intussusception in adults is surgical because of the high incidence of underlying malignant pathology and serious complications that can develop as a result of intestinal obstruction and vascular strangulation [7,11]. Most surgeons agree that resection is necessary, particularly in colonic intussusceptions and in older patients, because of the possibility of a malignant tumor [3,5,9,15,20,21]. It remains debatable whether reduction of the intussuscepting lesion should be attempted during an operation or whether 'en bloc' resection should be carried out without attempting reduction [9,15,21]. Previous reports advocated reducing the intussusception before resection [22,23]. Some authors have recommended a selective approach to resection, depending on the site of intussusception, which influences the type of pathology [12,15]. Chang and colleagues [24] (2007) recommended operative reduction for small-bowel intussusceptions but not for colonic intussusceptions. Gupta and colleagues [25] (2011) reported resection in 70% of colonic intussusceptions. The potential disadvantages of this approach are intraluminal seeding and tumor dissemination via venous flow, perforation and seeding of infection and tumor cells into the peritoneal cavity, and increased risk of anastomotic complications [26]. The advantages of intraoperative reduction of the intussusception prior to resection, especially when the small bowel is affected, are that it may preserve a considerable length of bowel and thereby prevent development of short-bowel syndrome. Begos and colleagues [15] are proponents of resection without attempting reduction when the bowel is inflamed, ischemic, or friable and in obvious colocolic intussusception (with the high likelihood of malignancy). In all other cases, reduction should always be attempted initially. In the present case, intraoperative findings indicated that a large length of small bowel was intussuscepted into ileoileo and cecocolic intussusception with vascular changes in the wall of the colon. So to preserve as much viable small bowel as possible, we made a round incision in the ascending colon and pushed proximally (backward) the cecum with the terminal ileum (Figure 3). Then after a checking for bowel viability, we performed a right hemicolectomy with resection of a long segment of the ileum with subsequent creation of primary single-layer anastomosis between the ileum and transverse colon.

The postoperative complication rate in adult intussusceptions is still reported by some authors [12,24] to be relatively high. Although there is no existing research on a large group of patients, complications are much more a consequence of missed diagnosis and delayed treatment than the result of anastomotic problems, according to current studies [7,12,24]. Yakan and colleagues [12] (2009), in their retrospective study, reported a 20% postoperative complication rate and a perioperative death rate of 5% due to severe sepsis complicated by multiple organ failure six days after the operation, but there was no leak of anastomosis. Also, Chang and colleagues [24] (2007) reported a postoperative death rate of 5.5% in adult intussusceptions treated surgically. The postoperative period was associated with serious complications in our case as well. However, thanks to multidisciplinary active treatment, our patient was discharged from the hospital in good condition on the 30th postoperative day.

In conclusion, the diagnosis of intussusception in adults can be difficult because of atypical and episodic symptoms. It is very important to intervene surgically early on, something that was not done in this case. A high level of clinical suspicion and an abdominal CT scan are most useful tools for making a timely diagnosis.

## Conclusions

This case, as well as a review of the literature, showed that a missed initial diagnosis of intestinal intussusception in adults can delay proper treatment and cause serious consecutive complications. Therefore, early surgical treatment is needed regardless of the etiology.

## Abbreviation

CT: computed tomography.

## Consent

Written informed consent was obtained from the patient for publication of this case report and any accompanying images. A copy of the written consent is available for review by the Editor-in-Chief of this journal.

## Competing interests

The authors declare that they have no competing interests.

## Authors' contributions

ASK and ARH performed surgery, analyzed and interpreted the patient data, and were major contributors in writing the manuscript. LMS performed surgery. SAK analyzed and interpreted the patient data and was a major contributor in writing the manuscript. LHGL and FIK performed the histological examination of the specimen. All other authors contributed equally to the manuscript. All authors read and approved the final manuscript.

## References

[B1] Hunter J Palmer JF On introsusception (read Aug 18, 1789) The Works of John Hunter, FRS London 1837 London: Longman, Rees, Orme, Brown, Green, Longman 587 593

[B2] Hutchinson J A successful case of abdominal section for intussusception Proc R Med Chir Soc 1873 7 195 198 10.1177/095952877405700106 PMC2150446 20896433

[B3] Eisen LK Cunningham JD Aufses AH Jr Intussusception in adults: institutional review J Am Coll Surg 1999 188 390 395 10.1016/S1072-7515(98)00331-7 10195723

[B4] Felix EL Cohen MH Bernstein AD Schwartz JH Adult intussusception; case report of recurrent intussusception and review of the literature Am J Surg 1976 131 758 761 10.1016/0002-9610(76)90196-3 937658

[B5] Balik AA Ozturk G Aydinli B Alper F Gumus H Yildirgan MI Basoglu M Intussusception in adults Acta Chir Belg 2006 106 409 412 17017694 10.1080/00015458.2006.11679917

[B6] Tan KY Tan SM Tan AG Chen CY Chang HC Hoe MN Adult intussusception: experience in Singapore ANZ J Surg 2003 73 1044 1047 10.1046/j.1445-2197.2003.t01-22-.x 14632903

[B7] Wang N Cui XY Liu Y Long J Xu HY Guo RX Guo KJ Adult intussusception: a retrospective review of 41 cases World J Gastroenterol 2009 15 3303 3308 10.3748/wjg.15.3303 19598308 PMC2710788

[B8] Peh WC Khong PL Lam C Chan KL Saing H Cheng W Mya GH Lam WW Leong LL Low LC Ileoileocolic intussusception in children: diagnosis and significance Br J Radiol 1997 70 891 896 9486064 10.1259/bjr.70.837.9486064

[B9] Azar T Berger D Adults intussusception Ann Surg 1997 226 134 138 10.1097/00000658-199708000-00003 9296505 PMC1190946

[B10] Agha FP Intussusception in adults AJR Am J Roentgenol 1986 146 527 531 3484870 10.2214/ajr.146.3.527

[B11] Zubaidi A Al-Saif F Silverman R Adult intussusception: a retrospective review Dis Colon Rectum 2006 49 1546 1551 10.1007/s10350-006-0664-5 16990978

[B12] Yakan S Caliskan C Makay O Denecli AG Korkut MA Intussusception in adults: clinical characteristics, diagnosis and operative strategies World J Gastroenterol 2009 15 1985 1989 10.3748/wjg.15.1985 19399931 PMC2675089

[B13] Yalamarthi S Smith R Adult intussusception: case reports and a review of literature Postgrad Med J 2005 81 174 177 10.1136/pgmj.2004.022749 15749793 PMC1743231

[B14] Constanzo A Patrizi G Cancrinni G Fiengo L Toni F Solai F Arcieri S Giordano R Double ileo-ileal and ileo-cecocolic intussusception due to submucous lipoma: case report G Chir 2007 28 135 138 17475113

[B15] Begos DG Sanor A Modlin IM The diagnosis and management of adult intussusception Am J Surg 1997 173 88 94 10.1016/S0002-9610(96)00419-9 9074370

[B16] Erkan N Haciyanli M Yildirim M Sayhan H Vardar E Polat AF Intussusception in adults: an unusual and challenging condition for surgeons Int J Colorectal Dis 2005 20 452 456 10.1007/s00384-004-0713-2 15759123

[B17] Hurwitz LM Gertler SL Colonoscopic diagnosis of ileocolic intussusception Gastrointest Endosc 1986 32 217 218 10.1016/S0016-5107(86)71810-5 3721141

[B18] Bar-Ziv J Solomon A Computed tomography in adult intussusception Gastrointest Radiol 1991 16 264 266 10.1007/BF01887362 1879648

[B19] Montali G Croce F De Pra L Solbiati L Intussusception of the bowel: a new sonographic pattern Br J Radiol 1983 56 621 623 10.1259/0007-1285-56-669-621 6883029

[B20] Lande A Schechter LS Bole PV Angiographic diagnosis of small intestinal intussusception Radiology 1977 122 691 693 841052 10.1148/122.3.691

[B21] Kitamura K Kitagawa S Mori M Haraguchi Y Endoscopic correction of intussusception and removal of a colonic lipoma Gastrointest Endosc 1990 36 509 511 10.1016/S0016-5107(90)71128-5 2227328

[B22] Donhauser DL Kelly EC Intussusception in the adult Am J Surg 1950 79 673 677 10.1016/0002-9610(50)90333-3 15410943

[B23] Brayton D Norris WJ Intussusception in adults Am J Surg 1954 88 32 43 10.1016/0002-9610(54)90328-1 13158719

[B24] Chang CC Chen YY Chen YF Lin CN Yen HH Lou HY Adult intussusceptions in Asians: clinical presentations, diagnosis, and treatment J Gastroenterol Hepatol 2007 22 1767 1771 10.1111/j.1440-1746.2007.04907.x 17914948

[B25] Gupta RK Agrawal CS Yadav R Bajracharay A Sah PL Intussusception in adults: institutional review Int J Surg 2011 9 91 95 10.1016/j.ijsu.2010.10.003 20951844

[B26] Marinis A Yiallourou A Samanides L Dafnios N Anastasopoulos G Vassiliou I Theodosopoulos T Intussusception of the bowel in adults: a review World J Gastroenterol 2009 15 407 411 10.3748/wjg.15.407 19152443 PMC2653360

